# Correction: Coping with COVID-19: Differences in hope, resilience, and mental well-being across U.S. racial groups

**DOI:** 10.1371/journal.pone.0320553

**Published:** 2025-03-21

**Authors:** Carol Graham, Yung Chun, Bartram Hamilton, Stephen Roll, Will Ross, Michal Grinstein-Weiss

The fifth author’s first name is incorrect. The correct name is: Will Ross.
