# Supplementary material for: Coping with COVID-19: Differences in hope, resilience, and mental well-being across U.S. racial groups
Source: PLoS One. 2022 May 19;17(5):e0267583. doi: 10.1371/journal.pone.0267583 (PMC9119465; doi:10.1371/journal.pone.0267583)
Supplement: S1 Appendix — (DOCX) [file pone.0267583.s001.docx]

Appendix. Survey Items Used in the Analysis

| ***Dependent Variables*** |  |
| --- | --- |
| Cantril Ladder | Please imagine a ladder with steps numbered from zero at the bottom to 10 at the top. The top of the ladder represents the best possible life for you and the bottom of the ladder represents the worst possible life for you. (0-10 Scale) |
|  | - On which step of the ladder would you say you personally feel you stand at this time? - On which step do you think you will stand about 5 years from now? |
| Mental Health | In general, how is your mental health currently, and how was your mental health 3 months ago and before the COVID-19 pandemic (before March 13, 2020)? (Excellent / Very good / Good / Fair / Poor ) |
|  | - Mental health currently |
|  | - Mental health 3 months ago |
| Social Distancing | How well do each of the following describe your "social distancing" behaviors since the start of the COVID-19 pandemic (March 13, 2020)? (0-100 Scale) |
|  | - I did not attend social gatherings |
|  | - If I had exhibited symptoms of sickness, I would have immediately informed the people around me |
|  | - I wore a mask to cover my face in public |
| COVID-19 Fear | On a scale of 0 to 100, where 0 is "not afraid at all" and 100 is "very afraid", how afraid are you of the COVID-19 pandemic? (0-100 Scale) |
| ***Independent Variables*** |  |
| Race | What race(s) do you consider yourself? Select all that apply: |
|  | - White/Caucasian |
|  | - Black/African American |
|  | - Asian |
|  | - Native American/Pacific Islander |
|  | - Other (please specify): ________________________________________________ |
|  | - Prefer not to say |
| Ethnicity | Do you consider yourself Hispanic or Latino/a/x? |
|  | - Yes |
|  | - No |
|  | - Prefer not to say |
| 2019 Household Income | In 2019, what was the total pre-tax income your household received from all sources, such as wages, Social Security, pensions, annuities, or side jobs? If you don't know the exact amount, give us your best estimate. _____________________________________________ |
| ***Control Variables*** |  |
| Age | What is your age? |
|  | ______________________ |
| Marital Status | Which best describes your marital status? |
|  | - Single, never married |
|  | - Married |
|  | - Separated |
|  | - Divorced |
|  | - Widowed |
|  | - Prefer not to say |
| Partner Status | Do you live with a partner? |
|  | - Yes |
|  | - No |
|  | - Prefer not to say |
| Gender | What gender do you identify as? |
|  | - Male |
|  | - Female |
|  | - A gender not listed here ________________________________________________ |
|  | - Prefer not to say |
| Number of Children | How many children under the age of 18 live in your household? |
|  | - 0 |
|  | - 1 |
|  | - 2 |
|  | - 3 |
|  | - 4 - 5 |
|  | - More than 5 |
|  | - Prefer not to say |
| Lost Job due to COVID-19 (Respondent) | Have you lost a job or lost income as a result of the COVID-19 pandemic? |
|  | - Yes |
|  | - No |
| Lost Job due to COVID-19 (Spouse/Partner) | Has your spouse or partner lost a job or lost income as a result of the COVID-19 pandemic? |
|  | - Yes |
|  | - No |
| Hardships during the Pandemic | Was there a time in the past 3 months when you or someone in your household: (Yes / No) |
|  | - Did not pay the full amount of the rent or mortgage because you could not afford it? |
|  | - Skipped paying a bill or paid a bill late due to not having enough money? |
|  | - Needed to see a doctor or go to the hospital but did not go because you could not afford it? |
|  | - Could not fill or postponed filling a prescription for drugs when they were needed because you could not afford it? |
| Health Insurance | What kind of health insurance do you currently have? If you have multiple forms, please select the one you would use for a trip to the emergency room. |
|  | - I am currently uninsured |
|  | - Insurance through employer |
|  | - Insurance through parent, spouse, or other family member |
|  | - Student insurance (through school / college) |
|  | - Private insurance (including bronze, silver, gold, and platinum plans sold on healthcare.gov or state insurance exchanges) |
|  | - Medicaid (typically for low-income households) |
|  | - Medicare (typically for persons 65+ years old or with a disability) |
|  | - Veteran's, retired, or current service personnel insurance (VA, Tricare, VHA, etc.) |
|  | - The Indian Health Service (IHS) or other government program (9) |
|  | - Other (please specify): ________________________________________________ |
| Vaccination Status | Have you been vaccinated for COVID-19?   - I have received one dose of the COVID-19 vaccine - I have received both doses of the COVID-19 vaccine - I have not received any doses of the COVID-19 vaccine |
| Vaccination Likelihood (if not vaccinated) | When the vaccine for COVID-19 becomes available to you, how likely are you to get vaccinated?   - Extremely likely - Somewhat likely - Neither likely nor unlikely - Somewhat unlikely - Extremely unlikely |
| COVID-19 Symptoms | Have you or anyone in your household had any symptoms (e.g., fever, dry cough, shortness of breath, aches and pains) that you believe were due to COVID-19? |
|  | - Yes |
|  | - No |
|  | - Prefer not to say |
| Educational Attainment | What is the highest level of education you have completed? |
|  | - Less than high school degree |
|  | - High school degree or G.E.D. |
|  | - Some college but no degree |
|  | - Certificate or technical degree |
|  | - Associate's degree |
|  | - Bachelor's degree |
|  | - Some graduate or professional school |
|  | - Graduate or professional degree |
